# The visible subtitler: Blockchain technology towards right management and minting

**DOI:** 10.12688/openreseurope.15166.1

**Published:** 2023-02-02

**Authors:** Pilar Orero, Anna Fernandez Torner, Estel·la Oncins

**Affiliations:** 1 Translation, Interpretation, and Eastern Asian Studies, Universitat Autonoma of Barcelona, Bellaterra, Barcelona, 08193, Spain

**Keywords:** subtitles, copyright, traceability

## Abstract

**Background: **Subtitles are produced through different workflows and technologies: from fully automatic to human in open source web editors or in-house platforms, and increasingly through hybrid human-machine interaction. There is little agreement regarding subtitle copyright beyond the understanding that it is a derivative work. While same language verbatim subtitles may have little room for creativity, interlingual subtitling is heavily dependent on the subtitler skills to translate, prioritise, and condense information. These days creative subtitles are increasingly being used as one more aesthetic element in audiovisual narrative. Though they may be in the same language, the visual attributes that contribute to the narrative development make creative subtitles one more element that should be acknowledged and copyright protected.

**Methods: **The paper will present a short introduction to subtitling copyright. It will then describe centralised and decentralised copyright management — where blockchain technology can be applied to aid subtitler identification. A focus group with expert professional subtitlers was organised, and feedback is reported.

**Conclusions: **Subtitle copyright is country dependent, still subtitling working practices and media asset distribution have no geographical borders. Blockchain technology -as a concept- could aid subtitle traceability. This can be useful beyond financial and moral right management and work towards media sustainability, allowing for reuse and repurpose of existing media assets.

## Introduction

There is no international copyright law that protects subtitles. Recent international treaties governing copyright law are: the Berne Convention for the Protection of Literary and Artistic ^1^ (1886), the Rome Convention ^2^ (1961), the Scientific and Cultural Organisation ^3^ (UNESCO) (1972), the TRIPS Agreement of the World Trade Organisation ^4^ (WTO) (1994), the Paris Convention of the United Nations Educational, Scientific and Cultural Organisation ^5^ (UNESCO) (1972), and the WIPO copyright treaty and the WIPO Performances and Phonograms Treaty ^6^ (1996), the Beijing Treaty on Audio-visual Performances ^7^ (2012) and the Marrakesh Treaty to Facilitate Access to Published Works for Persons who are Blind, Visually Impaired, or otherwise Print Disabled ^8^ (2013).

All these treaties include a framework for copyright based on principles, standards and rules for the protection of creative works and the rights of their authors, translators included. Yet, none of them provide a list of work genres or formats which can be protected under copyright law. For example, there is no mention of song lyrics in subtitles for television. Right management legislation is country dependent, and there are significant differences in copyright protection between countries worldwide ( Fishman, 2020).

Existing legislation in the US began the trend of compulsory subtitling media content as stated in the Section 508 ^9^ which is an amendment to the U.S. Rehabilitation Act that requires federal agencies to develop, procure, and maintain Information and Communication Technology (ICT) – including websites – that are accessible. The famous lawsuit in 2012 of the National Association of the Deaf vs Netflix set a landmark precedent ^10^. The case is interesting not only because subtitling became compulsory in the US, but also because of copyright issues. In the lawsuit Netflix also argued for dismissal because they were not the owner of the content copyright, nor the subtitles as derivative of the content. This exemption from the obligation to provide subtitles as requested by the 21st Century Communications and Video Accessibility Act ^11^ was also dismissed since it was found that “Netflix “owns, leases…, or operates”. The accessibility responsibility was fixed to distributors, and not to exhibitors (cinemas), film producers, nor the film director. The same US legislation -21st Century Communications and Video Accessibility Act- applies to educational content. These days there is no doubt any digital content should be subtitled (Section 508) ^9^. As the information from the Antelope Valley College ^12^, there are clear problems regarding how to generate subtitles and the copyright ownership. The issue with copyright is quite complex since subtitles are a text but shown as an image. The issue is also that anyone can download a subtitle file and make changes: edit, improve, change colour, type, font, size, ( Lasar, 2011; Tushnet, 2012) and upload it to the web claiming authorship. Subtitles may be translated, but some are a verbatim transcription, such as the lyrics of music videos ( Stanton, 2015). Producing subtitles is nowadays a complex exercise. They can be produced by one human — or a team. It may be produced by a speech to text engine and edited by a human, by crowdsourcing ( Matas, 2020), or 100% by a machine ( Stanton, 2015).

Under European copyright law, rights are endowed to the physical person, the creator (Directive 2004/48/EC of the EU parliament and the council of 29 April 2004 ^13^). This implies that an organisation or a business may only obtain the copyright through a transfer or a legal contract ( Nikolic & Bywood, 2021). This is again a challenge to companies who generate subtitles through proprietary automatic subtitling editors but can’t claim rights to protect from further uses. This has been identified and reported in the “Study on copyright and new technologies: copyright data management and artificial intelligence” ( European Union, 2022: 21) as “AI-generated output is not protected under copyright in the absence of human creative choices”. Right management is also difficult when a work is created by more than one person, for example through co-creation when several people collaborate leading to joint authorship (the resulting work is inseparable) or collective works, when they are separable, or when it is not created by a person at all but from AI for example ( Matamala & Soler-Vilageliu, 2022; Oncins forthcoming). 

## Subtitle copyright management

While the copyright management of translation of literary texts is legally recognised and studied extensively in the field of Translation Studies ( Bassnett & Bush, 2006; Federici, 2011; Gentzler, 1993; Venuti, 1982), little is known about the copyrights management in the translation audiovisual media assets ( Matamala & Orero, 2007; Matamala & Orero, 2019; Orero, 2005; Orero, 2007; Remael *et al.,* 2019). Subtitles experienced a transformation with the approval of the United Nations Convention of Rights of Persons with Disabilities (CRPD). CRPD is a Human Rights treaty adopted on 13 December 2006 during the sixty-first session of the General Assembly by resolution A/RES/61/106. In accordance with its article 42, the Convention was open for signature by all States and by regional integration organisations at United Nations Headquarters in New York as of 30 March 2007. In 2010, the European Union (EU) became a Party to the CRPD with the aim of promoting and protecting human rights of all persons and upholding democracy and the respect of the rule of law. The Council of Europe adopted two strategies named Union of Equality 2010–2020 ^14^ and 2021–2030 ^15^ “which paved the way to a barrier-free Europe and to empower persons with disabilities so they can enjoy their rights and participate fully in society and economy.” This had a direct impact on subtitling, as an accessibility service to any spoken media asset. New EU legislation such as the European Accessibility Act (Directive 2019/882 ^16^) and the Audiovisual Media Service Directive (Directive 2018/1808 ^17^) ( Orero forthcoming) will request 100% subtitling on any media content by 2025. What the directives don’t specify is the type of subtitles required: translations or transcriptions. In Europe subtitles have been traditionally defined as the translation of dialogues, such as movies, and they hold copyright (Directive 2019/790 ^18^ of the EU parliament and of the council on copyright and related rights in the Digital Single Market and amending Directives 96/9/EC and 2001/29/EC). CRPD triggered the use of a new type of subtitles: same language subtitles or subtitles for the deaf and hard of hearing ( Neves, 2005; Neves, 2008), and these subtitles are not considered original work, since they are transcriptions of the dialogue. The issue is that subtitles for the deaf and hard of hearing codifies in writing more information beyond the dialogue, such as sounds, emotions or music ( Rashid *et al*., 2006; Rashid *et al*., 2008; Rawsthorn, 2007; Romero-Fresco, 2013; Romero-Fresco, 2021a; Romero-Fresco, 2021b; Rosenberg, 2007; Zdenek, 2018). There could be the argument that this translation is creative, hence subtitles for the deaf and hard of hearing should also hold copyright — though they don’t.

Much work has been undertaken to understand the media accessibility obligations of the CRPD in Europe ( Morettini, 2014), the many steps in the subtitle production workflow by humans individually or in teams ( Sánchez, 2004), and the different professional profiles working in each step of the production chain ( Matamala, 2019). Technology is increasingly allowing new subtitling workflows from machine translation ( Nyberg & Mitamura, 1997) to crowdsourcing by humans as fansubs ( O’Hagan, 2009) to post editing machine translated subtitles ( Karakanta *et al*., 2022). Still we are in a cat-and-mouse-game: technology is incessantly developing new professional workflows to generate translated or transcribed subtitles, and trying to claim ownership to a complex creative process has many challenges.

This multiplicity of potential creators and copyright claimants from a textual format as Tushnet (2012: 26) explains for US:

“copyright begins with text: the Constitution speaks of the ―Writings of ―Authors. Conceptual maneuvers were required to allow copyright to cover all media. The official story is now one of media neutrality, except where specified otherwise. In the Copyright Act of 1976, Congress changed the definition of copyrightable works from ―all the writings of an author to ―original works of authorship. Nonetheless, the written text remains the prototypical copyrighted work. Perhaps judges, whose output is written, have a particularly easy time seeing the worth and creativity of writing, and analogizing other types of creation to words.”

## Centralised subtitle copyright management

Each EU country has its own copyright laws, and an agency to manage the rights. An example is the Spanish Law Ley de Propiedad Intelectual 1/1996 ^19^ ( BOE-A-1996-8930) and the agency DAMA ^20^ (Derechos de Autor de Medios Audiovisuales managing the subtitle rights. In turn the Spanish DAMA is a member if the worldwide agency CISAC ^21^ (International Confederation of Societies of Authors and Composers) who represent 228 member societies in 119 countries worldwide. The way they codify subtitles, as an audiovisual work, is through the ISAN (International Standard Audiovisual Number) as part of the International Standardisation Office ISO (ISO 15706:2002). This voluntary numbering system provides a globally unique identifier for each audiovisual work (ISAN 2005 ^22^). According to the International Confederation of Societies of Authors and Composers (CISAC):

“ISAN identifies works, not manifestations of the work such as publications or broadcasts. Thus, the assigned identifier remains the same irrespective of the various formats in which the work is distributed (video recording, DVD, *etc.*) or used. The ISAN identifier is agnostic to different language versions and thus supports the identification of audiovisual works across national boundaries and languages. When a film crosses borders, its title often changes. The ISAN identifier removes potential uncertainty with regard to different titles for the same film.
The ISAN identifier also facilitates more accurate and faster identification of audiovisual works with the automated processing of broadcast usage reports and the monitoring of Internet usages, thereby avoiding errors in the capture of data on the works. The ISAN system also plays a key role in tackling problems with piracy and the management of rights by member societies.”(CISAC ^23^)

ISAN highlights the many issues which are at stake when identifying the owner such as: formats, distribution, languages, countries, piracy, processing rights, use and reuse of the work. In a traditional subtitle, they are texts, and text files, hence copyright issues should be easier. While ISAN is the number used as identifier by national registration agencies, ISAN is not related to copyright registration “nor does the issuance of an ISAN provide evidence of the ownership of rights in an audiovisual work” (ISAN ^24^). The ISAN code identifies the work, and always relates and provides descriptive information about the audiovisual content.

Here is an example of an ISAN:

            ISAN 0000-0000-3A8D-0000-Z-0000-0000-6

The ISAN identifies works, not publications (unlike the International Standard Book Number ISBN for books) nor right holders. The ISAN remains the same for an audiovisual work regardless of the various formats in which the work is distributed ( *e.g.* DVD, video recording) or its use. The fact that subtitles may take many different forms, and on the screen they can be perceived as images complicates the matter, and for example features such as subtitle typeface and font can play an important role ( Neves, 2005). Tushnet (2012: 26) argues about paralinguistic elements of subtitles that “can even shaping meaning (look at the absolute hatred of many people for Comic Sans, or for messages transmitted in all capital letters), yet the Copyright Office has long refused to register copyrights in ―mere typographic variation”. The production of subtitles from: a human, a machine, human collaboratively, to post edition has also affected copyright ( Martin, 2002) and identifying the authorship of these new translation workflows and registering the copyright is increasingly complex.

The most popular model for subtitle right management is that professionals sign their exploitation rights to the subtitle agency or platform and are rarely updated with information regarding the ulterior uses or translations of the subtitle file. 

### Decentralised subtitle copyright management

During the last decades subtitlers work for large companies that have subtitle content creation and post edition and sharing as the basis of their business model. Platforms such as Amara ^25^, Rev ^26^, Happyscribe ^27^, Phrase ^28^, Gridly ^29^ or Netflix ^30^ currently dominate the subtitling market ( Widia *et al*., 2021). They have grown to the point of holding gate-keeping power and authority, and as a result they impose their own policies and terms in defining what type of copyright and right management is visible or even allowed to be shared and monetised ( Oziemblewska & Szarkowska, 2022). There are numerous other issues stemming from this centralisation of authority, including the demonetization of creative media content, opaqueness of decision making with respect to content moderation and the non-transparent and often unpredictable way in which these platforms permit third parties to integrate with them ( Foss, 2020).

This is a particularly important problem for subtitlers for a number of reasons: a) from a financial point of view, the price is fixed by the large digital platforms ( Foss, 2020); b) from a data protection point of view, there is very little control and accountability over how subtitlers data is managed by the large digital platforms ( Georgakopoulou, 2012) c) from a use and repurpose of subtitles point of view, there have been numerous incidents where the decisions and strategies of these platforms have unfairly disadvantaged subtitlers ( Kuscu-Ozbudak, 2022). The overall impact of the domination of the large digital platforms highlights the need for alternative platforms for creating and sharing media content that do not rely on central authorities, organisations or companies.

New digital technologies have been developed for decades now to avoid centralisation and to establish trustworthy alternatives to existing exchange systems, such as the Fiat monetary system ^i^ which is backed by governments and centralised for example in Europe by the European Central Bank. Blockchain is one of the technologies in the new decentralised digital ecology system. It began as part of the crypto economy as an “economic system, which is not defined by geographic location, political structure, or legal system, but which uses cryptographic techniques to constrain behaviour in place of using trusted third parties” ( Babbitt & Dietz, 2014, quoted in Pilkington, 2016: 228). The decentralised digital ecology gives rise to a new subset of transactions and finance which may be defined as “a formal discipline that studies protocols that govern the production, distribution and consumption of goods and services in a decentralized digital economy. Cryptoeconomics is a practical science that focuses on the design and characterization of these protocols” ( Zamfir, 2015) defines how it works:

“each agent is assigned a private key (kept secret like a password) and a public key shared with all other agents. A transaction is initiated when the future owner of the coins (or digital tokens) sends his/her public key to the original owner. The coins are transferred by the digital signature of a hash. Public keys are cryptographically generated addresses stored in the blockchain. Every coin is associated with an address, and a transaction in the crypto-economy is simply a trade of coins from one address to another. The striking feature of the blockchain is that public keys are never tied to a real-world identity. Transactions, although traceable, are enabled without disclosing one’s identity; this is a major difference with transactions in fiat currencies that, with the exception of (non-traceable) cash transactions, are related to specific economic agents endowed with legal personality (whether physical or juridical)”.

The positive side of a transaction using blockchain is that it is final once it is included in the blockchain, thereby becoming simultaneously verifiable by many sources ( Dwyer, 2014: 4). Applied to subtitling this would allow for its identification and for monetisation. There is also the need to endow a licence. For this the Distributed Ledger Technologies are a new possibility, using on the one hand a blockchain solution and Smart Contracts (SC) allow for notarising and executing transactions and on the other hand licences as Smart Legal Contracts (SLC) to meet the legal requirements.

SLCs are defined as a digital agreement ( Rühl, 2021). It consists of a natural language part and a machine-readable part with computational components. The human-readable part ensures that the different stakeholders (signatories, lawyers, *etc.*) can understand the contract, the machine-readable part allows the document to be interpreted and executed by computers ^iii^. In this way, SLCs enable subtitlers to provide tailored contracts to satisfy the client requirements, or to offer subtitles for free with an exploitation licence that at the end of the negotiation process is referenced on the blockchain to ensure their notarization.

## MediaVerse subtitle copyrights management framework

MediaVerse ^31^ is a three-year European funded project under the H2020 programme (2019-2022). The project aims to design and test a framework to allow professionals and laymen to publish multimedia content that may be easily shared and licensed and to empower media stakeholders to enjoy and produce inclusive, diverse, respectful, credible and accessible media experiences.

MediaVerse is looking at the legal aspects of copyright definition and will include the procedures for registering and storing in a common repository copyrights of content owners. The legal requirements for subtitles have been analysed from both professional subtitlers and legal experts, and a simplified set of rules/properties is proposed as a template for managing Intellectual Property (IP) across different organisations and countries. A machine-readable format will be defined to support the proposed legal framework which will allow the 1) representation, 2) storage/registration and 3) smart negotiation of (multimedia) content. These are according to the IP definition and licence chosen based on the services developed. This protocol enables a cross-border approach to copyright protection at a European level. Moreover, the approach implements all the procedures that a subtitler should follow to claim ownership of their work, as well as to provide licence suggestions —such as Creative Commons — for users uploading new content on the platform. The MediaVerse copyright repository is a prototype and only functions for providing information about content owners and copyrights. It is not intended to replace registration offices, which are required in some countries, nor is the repository intended to handle cases of copyright infringement.

The appeal of blockchain to subtitles can be seen in the possibility to render subtitle data that is reliable, immutable, transparent, and decentralised. The main advantages of blockchain in this perspective, is the guarantee of trust through peer-to-peer interactions without the need for a central governing body ( Qureshi & Megías Jiménez, 2012). Any person should be able to create and mint their subtitles.

MediaVerse, also offers the possibility of collaborative subtitle production and the collaborative right management. The co-authors have the possibility to agree on their respective roles towards the work. They can determine their identity as co-author or holder of a neighbouring right. MediaVerse will have no say in this process, nor will it check the attributed roles, putting power and contractual freedom in the hands of the MediaVerse users. As MediaVerse is available across European borders, the users will benefit from this definition of their roles and rights in respect to the work as the uncertainty of the application of diverging national laws in this regard will be excluded. Furthermore, the authors and holders of neighbouring rights can decide on their respective revenue shares in the MediaVerse licences. The user who uploads a collective or collaborative work in MediaVerse has full responsibility and warrants that the correct authorisations from the other right holders involved have been acquired. It is also possible to upload automatically generated subtitles that are not created by a person at all. Companies have the possibility to upload an automatically generated subtitle, claim ownership on it and licence the work within MediaVerse. MediaVerse thus allows the owner of an AI-system and a work generated by that system to share, sell and/or licence such work without the question of copyright protection. As the licence is a contract, it is enforceable also outside of MediaVerse regardless of the lack of copyright of the work. The implication here is that the owner will not have the specific enforcement possibilities which would be given to the author of a work under copyright law.

In MediaVerse IP rights management follows the concept of “triplets” where three elements are present: the subject, the right, and the object. Rights ( *e.g.,* ownership, copyrights, neighbouring rights, such as the right to reproduction and the right to make available to the public) are owned by a subject and refer to an object (a digital asset in the MediaVerse ecosystem). A “triplet” can refer to another “triplet” to represent dependencies between “rights”. For example, the right to use a subtitle by subject Y (which is obtained through a licence), will refer to the ownership right of subject X on the said subtitle. In this way we can both maintain the dependencies among the rights, as well as the possibility to always check the rights assignment.

The MediaVerse Blockchain Service Provider, that manages the Smart Contracts (SC) used to notarise the different instances of the Smart Legal Contracts, enabling the acquisition, and transferring of subtitle digital rights and supporting revenues sharing among the stakeholders. In MediaVerse, the blockchain is transparent to subtitlers who are able only to see their balance in their local fiat currency and the rights they own on specific digital assets. Different kinds of SCs will be used to manage rights.

MediaVerse Rights Management framework is designed to cover legal and technical aspects of intellectual property through a common digital rights management model, in particular by providing a machine-based legal framework to support the representation, registration, storage and negotiation of media content according to the relevant IP definition and associated licences. The framework was designed from requirements by professional subtitlers.

## Testing MediaVerse subtitle copyright management

The tools to validate the copyright management framework proposed by MediaVerse with users from the audiovisual translation field were initially tested in a focus group held in April 2022. The aim of the research was twofold. First, to gather and analyse data from users to understand the existing workflow for production, distribution, and monetisation of subtitles as accessibility media assets. Second, to gain information about subtitlers needs and expectations of the MediaVerse platform in relation to rights management of media accessibility assets from a user centric approach. Professional subtitlers were very interested in how this framework will develop further.

The objective of the focus groups was to reach a varied sample of experts from the audiovisual translation field (academia, industry, and research). Following the COnsolidated criteria for REporting Qualitative research (COREQ) based on Tong *et al*. (2007) we report the criteria used for demographics. The interviewer and facilitators are the authors of this paper, all females with PhD. The participants were three males professional subtitlers, two hold a BA and one a PhD, and two female professional subtitlers, one with a BA and one with a PhD. The relationship with participants were of having all shared teaching duties in a common MA in Audiovisual Translation at Universitat Autònoma de Barcelona (Spain). They were recruited *via* e-mail. The choice of recruitment was twofold: the experience as subtitlers of the major Hollywood film studies, and their participation as founding members of the Audiovisual Translation Association ATRAE ^32^, which protects subtitlers professional interests. The focus group duration was agreed with participants as 90 minutes and was held online using the Microsoft Teams video conference platform. At the end of the session, consent forms were digitally signed and obtained from all participants.

According to the procedure ( Orero, 2022a) following ( O’Brien *et al.,* 2014) first an introduction about the methodological orientation regarding the MediaVerse project and its aims was provided. The method of recording the participants comments were through note taking by one of the facilitators. No transcription was generated. The reasons for not video recording was a sustainability policy adopted by the researchers to avoid the carbon footprint that leaves the storage of a 90 minute video interview ( Kamiya, 2020; Travers, 2021). After the short introduction a theoretical presentation of blockchain technology as part of the MediaVerse platform to manage copyrights was explained through a set of slides in a PowerPoint Presentation. Finally a list of the possible blockchain-based solutions that could apply to the audiovisual translation sector was also presented as follows:

1. Decentralised digital content ecosystem: power and ownership return to creators.
2. New pricing options: new options for creators to earn by selling content.
3. Monetization of content: content creators can establish direct relationships with customers.
4. Distribution of royalty payments: near real time payments based on smart contracts.
5. From DRM (Digital Rights Management) to smart contract: Transparent and "self execute" right management underlying smart contracts.
6. Attribution: Blockchain increases the visibility and availability of the information regarding copyright ownership.
7. Copyright management: Blockchain enables content owners to directly manage their works.

Third, a discussion among participants was elicited by the facilitator and notes were hand taken by one designated note taker. The discussion was structured around the following three questions:

1. Would you use the MediaVerse platform in your professional context?
2. Within the frame of accessibility and audiovisual translation files ( *i.e,* media accessibility assets) rights management, authors have the moral right over the assets they create. This can never be sold. Thus, assets should be somehow minted for moral ownership. Do you agree?
3. Should authors be able to establish the economic rights and rights of exploitation

At the end of the focus group extracted conclusions for each question were read aloud and validated by all participants. The replies to the three questions were as follows.

### Use of the MediaVerse platform in the audiovisual translation sector

The five participants who are professional audiovisual translators work for cinema majors. Their work is performed on private platforms (Sfera, Pixelogic) to create subtitles. The participants reported on the workflow in the used subtitle platforms as follows: Once subtitles are created, they are locked, they are inaccessible, they cannot be revised or edited. Subtitlers are not allowed to have a copy of their own subtitles. Not all platforms are locked, some platforms like Netflix allow you to download and edit subtitles once created.

Since all participants work for majors with a centralised platform, they do not see the integration of blockchain in the near future. One of the reasons for such controlled centralised platforms is the high risk of piracy. Participants think it is unlikely majors will change their workflow for a decentralised platform such as that proposed by MediaVerse. Participants liked the concepts proposed by MediaVerse and suggested as a first step to dialogue with companies (especially majors) to allow subtitlers to work outside such platforms. They also suggested adding some of the MediaVerse platform functionalities, such as blockchain, to their existing platforms.

### Moral right. Within the frame of accessibility and audiovisual translation files (i.e, media accessibility assets) rights management, authors have the moral right over the assets they create. This can never be sold. Thus, assets should be somehow minted for moral ownership. Do you agree?

All participants agree that exploitation rights can be sold, but not moral rights, which cannot be waived. The issue raised here is the right management for accessibility services which do not generate rights (subtitles for the deaf and hard of hearing or audio subtitling) as they are not considered literary creations and there is no right to claim the authorship of such assets. Participants requested rights to all accessibility services, and the need to ask for permission to use it, regardless of the fact that you hold the exploitation rights for it or not. 

All participants also pointed out the collaborative nature of audiovisual translation from translation, lip synch, editor, *etc.* There are many processes. Participants shared concern about how to assign and identify authorship in the case of scripts for dubbing: translator + lip synchroniser. Suggestions were made:

a) A shared blockchain of co-authorship is proposed.
b) Two separate authors would need to be identified, as the products are different.

### Should authors be able to establish the economic rights and rights of exploitation

This is seen with scepticism. Monetising the assets seems to be unattainable for participants, in view of their experience they have signing contracts in relation to the financial exploitation of the assets they create.

## Conclusions

Media content production is nowadays a popular endeavour. Social media platforms such as Tik Tok ^33^ in their webpage is described “to capture and present the world’s creativity, knowledge, and precious life moments, directly from the mobile phone. TikTok enables everyone to be a creator, and encourages users to share their passion and creative expression through their videos.” The latest TikTok statistics show that, as of July 2022, the platform has over one billion monthly active users worldwide (DataReportal, 2022 ^34^). It also diminishes the rights management of the billion active content producers, since content producers cannot claim rights.

A similar situation is shared by amateur subtitlers, who have no claim over their subtitle moral and commercial rights. Subtitling is the only accessibility service enjoying copyrights, other services such as audio description or subtitles for the deaf and hard of hearing are not considered to be original work worth right protection according to copyright laws. Regardless of what the law considers to be a creative accessibility service, professional and non-professionals alike produce media assets. These can be registered and monetised, and media producers can trace their work, and decide over the licence for each item. While professional subtitle right management is centralised geographically to each national rights management agency, more users than ever are producing media assets either as individuals, or collectively — still their creative rights are not protected.

To make matters worse, the media content production workflow is complex, with translators, lip synchronisers, subtitle posteditors, *etc.* who all work on the same content. Tracking and protecting the work of the many professionals is again challenging. The article presented the possible solution to allow any media content producer, and especially subtitlers, to use a decentralised right management framework, along the results from a focus group organised to understand requirements for right management of professional subtitlers.

In a world that needs more than ever to care for the environment, minting media assets such as subtitles, will allow for their use, reuse and repurpose, avoiding duplication, and working towards a circular economy which hopefully will reduce the carbon footprint ( McDonagh & Orero forthcoming). There is a need to care for the planet, and media production, storage and streaming has an important impact on carbon emissions. As Traves (2021 ^35^) reports “To store and transmit all of the data powering the internet, data centres consume enough electricity to account for 1 percent of global energy demand — which is more than the total consumption for many countries. Even before the pandemic, the internet’s carbon footprint had been increasing and accounted for about 3.7 percent of global greenhouse gas emissions.”. Now more than ever we need to think creatively on ways to reduce media asset proliferation. We believe that a small step will be to think of a traceability system for subtitles, which is not as energy intensive as blockchain. This was the aim of the article, to report on the positive impacts of traceable subtitles on the professional and on the environment. 

## Ethics and consent

Ethic clearance was obtained by the Ethics Committee on Animal and Human Experimentation (CEEAH) of the Universitat Autònoma de Barcelona with the reference no. CEEAH 5207 and entitled "MEDIAVERSE" presented by Estel la Oncins Noguer on 10-30-2020.

## Data Availability

Zenodo: Focus Group for Block Chain for Subtitles. https://doi.org/10.5281/zenodo.7313420 ( Orero, 2022b) This project contains the following underlying data: PP-UAB-4_report.pdf (Completed notes) Data are available under the terms of the Creative Commons Attribution 4.0 International license (CC-BY 4.0).
