# Supplementary material for: The visible subtitler: Blockchain technology towards right management and minting
Source: Open Res Eur. 2023 Feb 2;3:26. [Version 1] doi: 10.12688/openreseurope.15166.1 (PMC10446087; doi:10.12688/openreseurope.15166.1)
Supplement: Peer review history

# Peer review: Reviewer response for version 1

**DOI:** 10.21956/openreseurope.16405.r33231

**Published:** 2023-07-17

**Authors:** Iris Serrat-Roozen

**Affiliations:** 1 Universidad Internacional de Valencia, Valencia, Valencian Community, Spain

The paper effectively addresses a significant gap in the translation field, specifically focusing on the complex relationship between copyright management and subtitling. With the constant evolution of new technologies impacting subtitling workflows, blockchain enables the transition from a centralized to a decentralized system. The article introduces the MediaVerse platform, which provides a decentralised tool for copyright management in the digital environment and allowing content creators to protect and track their works while enabling their use, reuse, and repurpose.

 The topic explored in this paper is both highly interesting and necessary. The proposed solution for an Intellectual Property Rights (IPR) management tool is innovative and provides a solution to the challenges posed by the evolving digital ecosystems. Moreover, the paper serves as a valuable resource for raising awareness among content creators, including both professional and amateur translators, about the importance of keeping control over the digital assets they create.

 Although the platform was tested in a focus group and despite being a small-case study it presents compelling ideas. However, it would be advisable to expand the number of focus groups to include the other AVT modalities and professionals in the industry. In addition, including a section to section discuss the limitations of the study and potential biases would be beneficial.

 Overall, the paper has the potential to stimulate discussion and contribute to the creation of a more sustainable, democratic and equitable web.

If any, are all the source data and materials underlying the results available?

Yes

Is the work original in terms of material and argument?

Yes

Is the work clearly and cogently presented?

Yes

Is the argument persuasive and supported by evidence?

Yes

Does the research article contribute to the cultural, historical, social understanding of the field?

Yes

Does it sufficiently engage with relevant methodologies and secondary literature on the topic?

Yes

Reviewer Expertise:

Accessibility, audiovisual translation and language learning

I confirm that I have read this submission and believe that I have an appropriate level of expertise to confirm that it is of an acceptable scientific standard.

# Peer review: Reviewer response for version 1

**DOI:** 10.21956/openreseurope.16405.r32177

**Published:** 2023-07-06

**Authors:** Catalina Jiménez-Hurtado

**Affiliations:** 1 Universidad de Granada, Granada, Andalusia, Spain

The article focuses on the copyright of subtitles (and other form of audiovisual translation processes) and describes the centralised and decentralised management of copyright. We understand that blockchain technology can help among others to improve the identification of authors and thus their rights.

 The article is very interesting and innovative. In addition, it contributes to improving the working conditions of professionals who are effectively exploited by the large enterprises and platforms and to dignifying their profession. To have created a discussion group with professionals with so much experience is adequate and very useful. However, I believe that the results could have been enriched if the study had been extended with two other working groups including different types of professionals, representatives of platforms, distributors, small companies in the sector. Another possibility to make the data collection more representative could have been the distribution of a questionnaire adapted to the different sectors: platform representatives, professionals, intermediaries, television channels, etc. In any case, the article is very interesting, and I am sure that these proposals have already been made or are being made within the framework of the project presented here.

If any, are all the source data and materials underlying the results available?

Yes

Is the work original in terms of material and argument?

Yes

Is the work clearly and cogently presented?

Yes

Is the argument persuasive and supported by evidence?

Yes

Does the research article contribute to the cultural, historical, social understanding of the field?

Yes

Does it sufficiently engage with relevant methodologies and secondary literature on the topic?

Yes

Reviewer Expertise:

Audio visual translation, accesibility, information management.

I confirm that I have read this submission and believe that I have an appropriate level of expertise to confirm that it is of an acceptable scientific standard.
